# Outcome of Endoprosthetic Hip Reconstruction Following Resection of Malignant Bone Tumors

**DOI:** 10.3390/cancers16162890

**Published:** 2024-08-20

**Authors:** Thilo Khakzad, Michael Putzier, Alp Paksoy, Daniel Rau, Leonard Thielscher, Nima Taheri, Silvan Wittenberg, Sven Märdian

**Affiliations:** Center for Musculoskeletal Surgery, Department of Orthopaedic Surgery, Charité—Universitätsmedizin Berlin, Charitéplatz 1, 10117 Berlin, Germanyalp.paksoy@charite.de (A.P.); sven.maerdian@charite.de (S.M.)

**Keywords:** megaprosthesis, outcomes, bone tumor, hip, tumor resection

## Abstract

**Simple Summary:**

This study explored the long-term outcomes of patients who underwent hip replacement surgery following the removal of malignant tumors in the hip area. The main focus was to understand how well patients recovered in terms of physical function and quality of life. The study included 30 patients treated between 2010 and 2018, all of whom received interdisciplinary therapy, consisting of both systemic and surgical treatments. The follow-up period averaged a little over three years. The results showed that younger patients tended to have better physical function and overall quality of life after surgery. The study also revealed that complications like implant failure were relatively low, and no amputations were necessary. These findings suggest that this type of surgery, combined with comprehensive interdisciplinary care, can be a safe and effective option for managing hip tumors. Further research is needed to continue improving patient outcomes and tailoring treatments to individual needs.

**Abstract:**

Introduction: Over the past few decades, tumor arthroplasty has evolved into an established therapeutic approach for addressing bone defects following tumor resection in the extremities. As the diagnosis has a significant impact on patients’ lives, it is important to give clear expectations for functional recovery. Therefore, we investigated both the functional outcomes and the quality of life (QoL) after tumor arthroplasty for malignant hip tumors. Methods: This retrospective study included patients who had undergone resections of malignant hip tumors with consecutive modular hip arthroplasty between 2010 and 2018. Demographics, tumor entity, and complications stemming from both tumors and treatments were evaluated through the analysis of medical records and perioperative records. The assessment of functional outcomes was conducted with the following patient-reported outcome measures (PROMs): the Harris Hip Score (HHS), Musculoskeletal Tumor Society Score (MSTS), and the Short Form Survey 36 (SF-36). Furthermore, we performed subgroup analysis in two groups: one divided into survivors and non-survivors, as well as younger individuals (<57 years) and older individuals (>57 years). Results: A total of 30 patients were included in the study. At the time of follow-up, 19 patients were deceased. The average duration of follow-up was 3.2 (±2.51) years. The average age at the time of surgery was 60.3 (±15.20) years. Notably, there were no cases of amputation reported (0%). Five cases of implant failure were identified (16.67%). Among these, one was attributed to infection (3.3%), while four resulted from aseptic loosening (13.3%). In terms of functional outcomes, MSTS indicated good results (18 ± 7; range: 7–28; 60%), and the HHS demonstrated moderate outcomes (75.3%). Younger survivors (<57 years) exhibited notably superior results in terms of both the MSTS and physical functioning in the SF-36 (*p* = 0.03). Conclusion: In summary, this study shows declining tumor arthroplasty-related complications and satisfying functional outcomes as well as QoL. Noteworthy aspects include the relatively low rates of amputation and local tumor recurrences, which significantly favor the selection of appropriate therapeutic options. Moreover, the findings underscore the substantial impact of patients’ age on overall functionality and engagement in daily activities.

## 1. Introduction

Over the past decades, the field of reconstructive surgery, particularly involving megaprosthesis, following bone tumor resection, has undergone continuous development and has firmly established itself as a preferred alternative to amputation [1,2]. Notably, primary amputation has become avoidable in over 90% of patients today. In conjunction with surgical advancements, an interdisciplinary approach to treatment involving neoadjuvant and adjuvant combinations of radiochemotherapy has been significantly correlated to more favorable outcomes for patients [3,4]. Therefore, surgical treatment is only opted for after thorough analysis as part of considerations by the interdisciplinary tumor boards [3,4]. Due to improved and prolonged patient survival contributing to increased stress on prostheses, higher biomechanical demands are being imposed. Despite substantial improvements in prosthesis durability through ongoing advancements, instances of revisionary interventions due to implant failures continue to occur [5,6]. Mechanical and infectious complications persist despite significant progress in the field. For instance, Henderson et al. discussed postoperative instability as being influenced by the chosen access route for implanting a hip tumor prosthesis [7]. Moreover, these same prostheses demonstrated enhanced functional outcomes when combined with additional ligamentous reconstruction procedures [8,9].

As the diagnosis of a hip tumor has a significant influence on a patient’s life, clear expectations regarding the functional outcomes as well as QoL after surgery are of upmost importance. Our study aims to quantify the long-term functional outcomes and health-related QoL after malignant hip tumor resection with consecutive tumor arthroplasty. We hypothesize that a younger age at the time of surgery is associated with significantly better outcomes.

## 2. Materials and Methods

### 2.1. Inclusion and Exclusion Criteria

Ethical approval was obtained from the ethics committee of the local university (EA1/048/22), and the study was conducted in accordance with the principles outlined in the Declaration of Helsinki. This study took place within a university clinic in Germany that possesses certification as a tumor and sarcoma center.

The study included individuals who had undergone resection of either a primary or secondary bone tumor in the hip region and subsequently received a tumor hip arthroplasty between 2010 and 2018 as part of an interdisciplinary treatment approach. All patients received a tumor prosthesis of the MUTARS design (MUTARS, Implantcast GmbH, Buxtehude, Germany) and were treated exclusively within the same facility, ensuring a high level of comparability within the study cohort.

Patients who appeared to have passed away but for whom no verifiable date of death could be confirmed, as well as those who refused to participate, were excluded from the study.

### 2.2. Follow-Up Examination

During the follow-up assessment, demographic data were recorded. For functional evaluation, we assessed range of motion (RoM), the Harris Hip Score (HHS), and the Musculoskeletal Tumor Society Score (MSTS). Health-related quality of life (QoL) was measured using the Short Form Survey-36 (SF-36), which includes an overall score and eight subscales: physical functioning (PF), role physical (RP), bodily pain (BP), general health (GH), vitality (VT), social functioning (SF), role emotional (RE), and mental health (MH). Only the 11 patients who survived the follow-up period were called back for assessment, and their functional scores and QoL were prospectively evaluated. Deceased patients were included in the baseline analysis if a date of death was available, with the date of death considered as the follow-up endpoint. We conducted a subgroup analysis to assess additional factors influencing outcomes. Group A was categorized by the need for revision surgery, and Group B was divided by age (≤57 years vs. >57 years).

We also evaluated the impact of pathological fractures and the distinction between primary and secondary tumors on survival, including all 30 patients in this retrospective analysis.

### 2.3. Surgical and Histopathological Data

Perioperative data (age at surgery, tumor type, and the incidence of revision surgeries) was analyzed, evaluating surgical and anesthesiologic reports. Furthermore, we screened the data for pathological fractures, amputations, and time of death.

### 2.4. Statistical Analysis

Statistical analysis was conducted using SPSS version 25 (SPSS Inc., Chicago, IL, USA). Descriptive statistics are presented as either numbers (percentage) or means (standard deviation). Categorical variables were assessed using Chi-square and Fisher’s exact test, while continuous variables were analyzed using *t*-tests and the Mann–Whitney U test. Survivorship was determined through Kaplan–Meier methodology. The threshold for significance was set at *p* < 0.05.

## 3. Results

### 3.1. Patient Characteristics

We identified 30 patients who underwent megaprosthesis implantation at the proximal femur. At the time of this study, 19 patients (63.3%) had passed away. The remaining 11 patients, comprising 6 females and 5 males, participated in the prospective clinical follow-up examination. The mean duration of follow-up was 3.2 (±2.5) years. The average age at the time of tumor prosthesis implantation was 60.4 ± 15.2 years (Table 1 and Table 2).

Sixteen patients (53.3%) had an underlying primary bone or soft tissue tumor as the malignant condition, while bone metastasis served as the etiology of the bone lesion in 14 instances (46.7%). Among the 30 patients assessed, 10 (33.3%) had experienced a pathological fracture before undergoing tumor arthroplasty. All 30 patients (100%) managed to avoid extremity amputation.

In terms of implant outcomes, we identified five cases of implant failure (16.6%). One of these cases was attributed to infection (3.3%, classified as Henderson type 4), while four cases stemmed from aseptic loosening (13.3%, classified as Henderson type 2). Throughout the observation period, there were no instances of dislocations, periprosthetic fractures, or tumor recurrence (Table 3).

Functional outcomes in patients not requiring revision surgery (A1) were notably superior to those requiring revision surgery (A2), as reflected by the HHS (*p* = 0.183), MSTS (*p* = 0.133), and KPS (*p* = 0.921) (Table 4). However, these differences did not reach statistical significance. Health-related QoL, as assessed by the SF-36, did not exhibit significant differences, neither in overall nor in subscale scores (*p* > 0.05).

Regarding the influence of age, patients younger than 57 years at the time of surgery (B1) achieved better outcomes in HHS, MSTS, and KPS (Table 5) compared to older patients (B2) but did not exhibit significant differences. MSTS scores were significantly better in younger patients (*p* = 0.03). Furthermore, QoL was better in younger patients considering overall score and nearly all subscales except for ER and MH subscales. Younger patients performed significantly better on the PF subscale (*p* = 0.03).

### 3.2. Association between Patient Characteristics and Survival

To examine the impact of comorbidities on patient outcomes, we utilized the Charlson Comorbidity Index (CCI). Patients that were deceased at follow-up had a significantly higher CCI value (*p* = 0.004). No substantial differences in survival were observed based on factors such as sex, tumor entity, type of arthroplasty, need for revision surgery and local or systemic cancer recurrence (*p* > 0.005).

**Figure 1:**
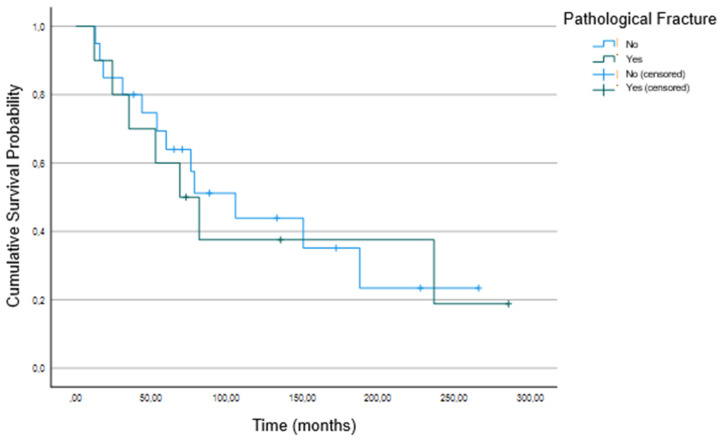
Kaplan–Meier curve of survival between the group with and without pathological fractures of all 30 participants.

**Figure 2:**
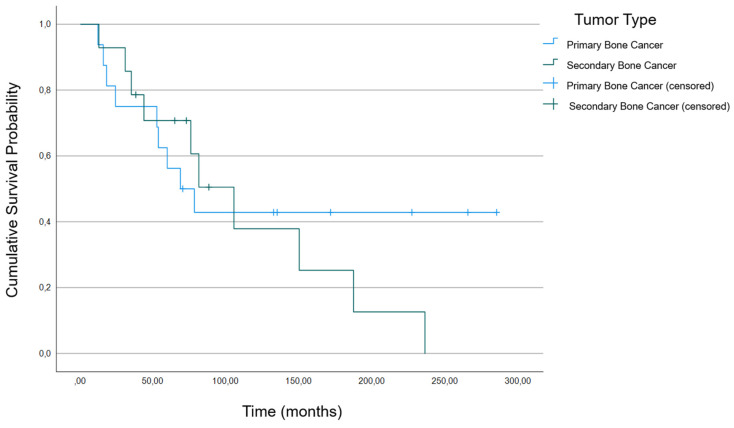
Kaplan–Meier curve of survival between the groups with primary and secondary bone tumors for all 30 participants.

Log-rank test did not reveal any significant decrease in survival associated with the incidence of pathological fractures (Figure 1, *p* = 0.805) or with the underlying tumor entity (Figure 2; *p* = 0.553).

### 3.3. Example Case

**Figure 3:**
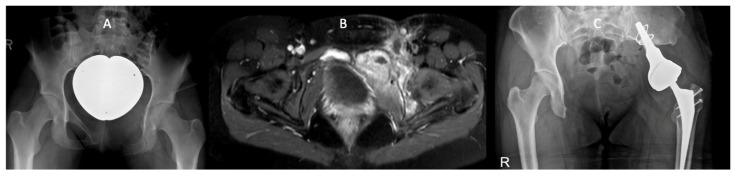
Example Case; Image (**A**) shows the preoperative finding of the Ewing sarcoma in a pelvis overview radiograph. Image (**B**) depicts the tumor spread in an axial MRI scan. Image (**C**) shows the latest pelvis overview radiograph after the implantation of the tumor endoprosthesis.

A female patient Figure 3 was diagnosed with an extensive PNET/Ewing sarcoma in the left anterior pelvic ring, extending supra-acetabularly and infiltrating the pubic bone on the opposite side. The tumor was classified as Enneking stage 2B. Following an interdisciplinary tumor board review, she initially received neoadjuvant polychemotherapy according to the Euro-Ewing protocol. Subsequently, a partial hemipelvectomy and resection of the anterior pelvic ring were performed, with the implantation of a tumor endoprosthesis (Mutars Lumic stem and cup, IC femoral head) and bladder suture. This was followed by adjuvant chemotherapy.

After 8.7 years of follow-up, the patient demonstrated positive outcomes in the study. Her functional scores were 49 (HHS) and 14 (MSTS), while her quality of life was assessed with an SF-36 score of 63 and a KPS score of 80.

## 4. Discussion

Our study aimed to investigate the factors influencing functional outcomes and quality of life (QoL) as well survival rates following a tumor prosthesis implantation of the hip. Our data suggest a trend that younger patients, as expected, tend to have better functional outcomes after implantation. However, this was not definitively proven in key tests such as the Harris Hip Score (HHS). The study also analyzed the impact of pathological fractures and tumor entity, categorized into primary and secondary tumors of the bone, on survival rates. While pathological fractures or the tumor entity did not significantly affect survival, our findings confirmed that tumor arthroplasty is a safe procedure with a lower incidence of complications compared to what is reported in the literature.

In comparison to the existing scientific literature, our patient cohort aligns well with the reported demographics. For instance, Janssen et al. showcased an average age range of 26 to 71 years across 33 studies encompassing a total of 1701 tumor prosthesis cases [10]. The distribution of underlying conditions leading to the need for tumor arthroplasty includes primary bone tumors in 37%, benign bone tumors in 4.1%, secondary bone tumors in 53%, and hematological diseases in 5.9% [10].

We demonstrated functional outcomes that were satisfactory to good. MSTS scores in our cohort were good with a score of 18 ± 7 points (range: 7–28; 60%). This outcome is consistent with the scientific evidence. Janssen’s systematic review reported MSTS scores ranging from 56% to 94% [10]. The diversity in results can be attributed to patient age, with younger individuals often achieving higher functional outcomes. This is exemplified by studies such as Khan et al., where a younger group with a mean age of 36 years achieved a notably higher MSTS score of 94% [11], whereas older cohorts exhibited lower scores, emphasizing the influence of age on functional outcomes. More recently, Liu et al. as well as Andreani et al. showed comparable MSTS scores at last follow-up with 22.25 ± 4.55 and 23.2 (range 10–30), respectively [12,13]. Andreani also showed an inverse linear correlation between the patients’ age and their post-operative functionality: younger patients had significantly better MSTS scores at the final follow-up of their study (*r*  =  −0.518; *p*  =  0.013) [13]. Regarding the Harris Hip Score (HHS), we reported average results, in line with values discussed in the literature ranging from 75% to 81% [14,15,16,17]. While a trend toward better HHS scores in younger patients was observed, the results did not reach statistical significance. It is generally postulated that a person’s overall condition, which is considered to be better in younger individuals, is the most important factor influencing physical and mental outcomes. The Harris Hip Score (HHS) is a well-established tool for analyzing functional outcomes even after primary hip prostheses for degenerative conditions. Typically, HHS values range between 80 and 90, as reported in the meta-analysis by Meermans et al., which compared 42 studies on different surgical approaches [18]. The expectedly poorer outcomes following tumor prosthesis implantation can be attributed to the larger resection area, removal of muscle attachments, and even the use of muscle attachment tubes.

A case report of a 16-year-old female also showed a satisfactory HHS score of 83 [19]. Zhao et al. compared MSTS scores of patients undergoing eggshell and non-eggshell procedures together with the implantation of megaprostheses and found the HHS to be between 83.6 ± 5.8, for the eggshell procedure, and comparable to our results without the eggshell approach, at 65.5 ± 11.9 points [20].

Notably, amputation of extremities was completely avoided in our cohort, indicating excellent results that align with the findings of Pennekamp and Janssen’s review [10,21].

In the context of implant failure, our study identified a rate of 13.3%, with infection accounting for 3.3% and aseptic loosening for 10%. This is comparable to the rates described by Henderson, Janssen, and others [3,10,19]. Aseptic loosening remains the most common cause of revision total hip arthroplasty, including those related to degenerative conditions [22]. Aseptic loosening is primarily caused by factors such as prosthesis design, surgical technique errors, wear particles from conventional polyethylene leading to osteolysis, inadequate fixation of components, and adverse local tissue reactions associated with metal ion concentrations. The influence of follow-up duration on implant failure rates is evident, with longer follow-up periods correlating with higher failure rates [6,17]. More recently, implant failure due to structural failure has been shown to be in the lower single digit area [23,24]. We were not able to confirm the high implant failure rates of 19% reported by Hobusch et al. [6]. The infection rates are comparable with recent literature reporting values between 6 and 19% [25,26,27]. Therefore, infection rates are generally higher than in primary hip arthroplasty, potentially due to extensive soft tissue damage, longer duration of surgery and high exposure to metal surfaces in combination with the potential tissue toxicity of (neo)adjuvant treatment [28].

Interestingly, our findings indicate that revision surgery did not significantly worsen functional outcomes. Health-related QoL using the SF-36, the overall score was 64.3%, with only younger patients showing significantly better scores on the PF subscale. The lack of association between implantation age and improved QoL may suggest that younger patients may experience both benefits and challenges from the tumor diagnosis with more intensity. They demonstrated inferior outcomes in terms of emotional role functioning (ER) and social role functioning (SR). This could be caused by fear of recurrence (FcR). Psycho-oncological studies have revealed a correlation between heightened stress levels, intensified therapeutic interventions, and compromised physical functioning, overall health, and mental well-being [29,30].

It is important to acknowledge the limitations of our study. The scarcity and diverse clinical, morphological, and histopathological heterogeneity encountered in primary and secondary bone tumors underscore the intricate complexity of this subject. Furthermore, our retrospective study design is intrinsically reliant on the quality of the data collection and retention, which at times might have led to the inadvertent loss of essential perioperative information.

Our study demonstrates satisfactory functional outcomes and health-related quality of life results and offers valuable insights into the outcomes of this approach. To enhance the evidence base, multicenter prospective studies focusing on individual tumor entities should be conducted to provide a more comprehensive overview.

## 5. Conclusions

Our study demonstrates satisfactory functional outcomes and health-related quality of life results and offers valuable insights into the outcomes of endoprosthetic reconstruction of the hip following the resection of malignant bone tumors. To enhance the evidence base, multicenter prospective studies focusing on individual tumor entities should be conducted to provide a more comprehensive overview.

## Figures and Tables

**Table 1:** Demographics for study participants; CCI = Charleston Comorbidity index, SD = standard deviation.

Patients (*n*)	30
Average age ± SD (range)	60.34 ± 15.20 (20–83)
Years of treatment	2010–2018
Sex male: female (*n*)	17:13
Age adjusted CCI ± SD (range)	6.4 ± 2.8 (2–10)

**Table 2:** Data of arthroplasty-related complications.

Follow-up (years)	3.16 (±2.51)
- Survival	4.62 (±2.82)
- Non-survival group	2.31 (±2.02)
Cancer-related death (*n*)	19
Number of patients with revision surgery (*n*)	5
- Infection	1
- Loosening	4
- One revision surgery	2 (2/2 loosening)
- Two revision surgeries	2 (½: 1. Loosening 2. Infection, ½: 1. Loosening 2. Loosening)
- Three revision surgeries	1 (1. Loosening 2. Infection 3. Infection)
Amputations (*n*)	0
Local cancer recurrence (*n*)	1
Systemic cancer recurrence (*n*)	8

**Table 3:** Characterization of underlying tumor conditions.

Tumor Type (*n*)	
- Osteosarcoma	1
- Chondrosarcoma	9
- Ewing Sarcoma	1
- Renal carcinoma metastasis	7
- Hepatocellular carcinoma	1
- Liposarcoma	1
- Uterine leiomyosarcoma	1
- Prostate cancer	1
- Breast cancer	2
- Non-small cell carcinoma	1
- Undifferentiated pleomorphic sarcoma	3
- Angiosarcoma	2
Pathological fracture (*n*)	10
Arthroplasty type (*n*): proximal femur	30

**Table 4:** Functional outcomes after implementation of tumor arthroplasty in the overall cohort.

PROM	Mean ± SD (Range)
HHS	75.3 ± 15.3 (49–96)
MSTS	18 ± 7 (7–28)
KPS	74.55 ± 16.95 (50–90)
SF-36 overall	64.3 ± 14.9 (33.2–88.6)
PF	50.45 ± 28.06 (15–90)
PR	61.36 ± 49.20 (0–100)
BP	61.64 ± 31.15 (10–100)
GH	58.64 ± 19.38 (25–85)
VIT	50.09 ± 16.4 (30–80)
SR	92.18 ± 15.03 (50–100)
ER	78.73 ± 30.95 (33–100)
MH	75.27 ± 14.06 (48–92)

**Table 5:** Functional outcomes after implementation of tumor arthroplasty for the respective subgroups.

	No Revision (A1)	Revision (A2)	*p*-Value	Age < 57 (B1)	Age > 57 (B2)	*p*-Value
Number	8	3	-	6	5	-
HHS	81.9 ± 11.0	60 ± 17.3	0.183	79.2 ± 20.2	71.4 ± 11.7	0.257
MSTS	20.0 ± 7.1	12.7 ± 3.2	0.133	20.5 ± 8.2	15.0 ± 4.3	0.030
KPS	75.0 ± 16.9	73.3 ± 20.8	0.921	80 ± 15.5	68 ± 17.9	0.052
SF-36 overall	0.68 ± 0.14	0.55 ± 0.19	0.279	0.69 ± 0.15	59 ± 0.16	0.247
PF	0.55 ± 0.29	0.38 ± 0.25	0.497	0.64 ± 0.26	0.34 ± 0.22	0.030
PR	0.72 ± 0.45	0.33 ± 0.58	0.376	0.79 ± 0.40	0.87 ± 0.30	0.126
BP	0.71 ± 0.26	0.37 ± 0.36	0.133	0.69 ± 0.32	0.53 ± 0.31	0.329
GH	0.61 ± 0.17	0.52 ± 0.28	0.630	0.63 ± 0.19	0.53 ± 0.21	0.537
VIT	0.52 ± 0.20	0.37 ± 0.16	0.133	0.56 ± 0.18	0.45 ± 0.17	0.662
SR	0.91 ± 0.17	0.96 ± 0.07	0.999	0.88 ± 0.19	0.98 ± 0.05	0.792
ER	0.79 ± 0.31	0.78 ± 0.39	0.999	0.72 ± 0.33	0.87 ± 0.30	0.792
MH	0.56 ± 0.16	0.71 ± 0.20	0.776	0.77 ± 0.11	0.74 ± 0.19	0.999

## Data Availability

The data presented in this study are available on request from the corresponding author.
